# Development of reference Image Quality Metrics for quantitative MRI research using MRIQC

**DOI:** 10.1002/alz70862_109959

**Published:** 2025-12-23

**Authors:** Himanshu Joshi, Aarthi G, Mekha S Thomas, Gowthami Nair, Sivakumar PT, Ganesan Venkatasubramanian, Paul M. Thompson, John P John

**Affiliations:** ^1^ Multimodal Brain Image Analysis Laboratory (MBIAL), National Institute of Mental Health and Neurosciences (NIMHANS), Bengaluru, Karnataka India; ^2^ Departmet of Psychiatry, National Institute of Mental Health and Neurosciences (NIMHANS), Bengaluru, Karnataka India; ^3^ National Institute of Mental Health and Neurosciences (NIMHANS), Bengaluru, Karnataka India; ^4^ Imaging Genetics Center, Mark and Mary Stevens Neuroimaging and Informatics Institute, Keck School of Medicine, University of Southern California, Marina del Rey, CA USA; ^5^ Multimodal Brain Image Analysis Laboratory (MBIAL), Department of Psychiatry, National Institute of Mental Health and Neurosciences (NIMHANS), Bengaluru, Karnataka India

## Abstract

**Background:**

MRI Quality Control (MRIQC) has emerged as a promising tool to assess the image quality of MR acquisitions using Image Quality Metrics (IQMs). The no‐reference IQMs for structural MRIs from the ABIDE (*n* = 1111; from 21 sites) dataset are available on the MRIQC documentation page. These IQMs consist of measures based on noise (cnr, snr), artifact detection (art_q1), information theory (efc, fber), and other measures (fwhm) related to the spatial distribution of image intensity. No standard reference IQM ranges are available in the literature as a guide for MRI researchers. Here, we report our study's preliminary results that aim to generate reference IQM ranges for quantitative MRI analyses using a randomly chosen subset of 100 scans from the ABIDE dataset.

**Method:**

The ratings of four raters under ‘OK’, ‘Maybe’, and ‘Fail’ were extracted without the IQMs for the randomly chosen subset of 100 scans from the MRIQC documentation of the ABIDE dataset. The 4^th^ rater was excluded as the ratings were unavailable for all the anatomical scans. Three trained raters from MBIAL (A.G., M.S.T. & G.N.) independently rated these 100 scans as above. We estimated the concordance of ratings between the ABIDE raters (*n* = 3) and MBIAL raters (*n* = 3). Then we calculated the average IQMs of scans that showed consensus amongst all 6 raters under ‘OK’ (*n* = 19), ‘Maybe’ (*n* = 0) and ‘Fail’ (*n* = 2) categories.

**Result:**

Visual inspection ratings of the ABIDE raters (Fleiss Kappa = 0.0325; z=5.63; *p* <0.001) and MBIAL raters (Fleiss Kappa =0.0401; z=9.62 *p* <0.0001) showed fair agreement. 19 scans were rated ‘OK’, and 02 scans were rated ‘Fail’ by all the six raters. The average IQM values for the 19 scans rated as ‘OK’ with perfect concordance between all the 6 raters were cnr=10.01±2.20, snr=15.50±3.82, art_qi1=0.06±0.03, efc=1.85±0.77, fber=15.93±17.49, fwhm= 3.48±0.62, while the corresponding values for the 02 scans rated as ‘Fail’ with perfect concordance were: cnr=5.21±0.35, snr=9.52±1.16, art_qi1=0.02±0.002, efc=0.44±0.04, fber=109.73±18.39, fwhm= 2.87±0.37.

**Conclusion:**

The proposed reference IQMs, once established in larger samples, have potential utility in developing automated QC pipelines for big data MRI analyses.

